# Satisfactory 2-year outcome of minimal invasive hybrid stabilization with double treated screws for unstable osteoporotic spinal fractures

**DOI:** 10.1007/s00068-024-02645-1

**Published:** 2024-08-30

**Authors:** Mohamad Agha Mahmoud, Anas Afifi, Maher Ghandour, Ümit Mert, Christian Herren, Christian Blume, Miguel Pishnamaz, Frank Hildebrand, Stavros Oikonomidis, Rolf Sobottke, Michel Teuben

**Affiliations:** 1 Department of Spine, Neuro- and Orthopedic surgery, Rhein-Maas Clinic, Würselen, Germany; 2 Department of Orthopedics, Trauma and Reconstructive Surgery, University Hospital RWTH Aachen, Pauwelsstr. 30, 52074 Aachen, Germany; 3 Department of Trauma and Orthopedic Surgery, Helios University Hospital Wuppertal, University of Witten/Herdecke, Wuppertal, Germany; 4 Department of Orthopeadic and Trauma Surgery, Faculty of Medicine and University Hospital Cologne, University of Cologne, Cologne, Germany; 5 Department of Traumatology, University Hospital Zürich, Zürich, Switzerland; 6 Department of Neurosurgery, University Hospital RWTH Aachen, Pauwelsstr. 30, 52074 Aachen, Germany

**Keywords:** Osteoporotic vertebral fracture, Hybrid stabilization, Minimally invasive

## Abstract

**Purpose:**

This study evaluates whether the fracture level alters the outcomes of minimally invasive hybrid stabilization (MIHS) with double-threaded, uncemented polyaxial screws for unstable osteoporotic vertebral fractures.

**Methods:**

This prospective cohort study included 73 patients (71.23% females, mean age: 79.9 ± 8.8 years) with unstable OF 3–4 fractures treated by MIHS between Nov 2015-Jan 2018. Patient characteristics, operative data, clinical outcomes, complications, radiological outcomes, and midterm (24-month) follow-up regarding functionality, pain, and quality of life were analyzed.

**Results:**

Patients had thoracolumbar (71.23%), thoracic (10.97%), and lumbar (17.8%) fractures. Operative time was < 120 min in 73.97% of patients, with blood loss < 500 ml in 97.25% of cases. No in-hospital mortality was recorded. Spine-associated complications occurred in 15.07% of patients, while 36.98% of patients had urinary tract infections (*n* = 12), pneumonia (*n* = 5), and electrolyte disturbances (*n* = 9). The mean length of hospital stay was 13.38 ± 7.20 days. Clinically-relevant screw loosening occurred in 1.7% of screws, and secondary adjacent fractures were diagnosed in 5.48% of patients. The alpha-angle improved significantly postoperatively (mean change: 5.4°) and remained stable for 24 months. The beta-angle improved significantly from 16.3° ± 7.5 to 10.8° ± 5.6 postoperatively but increased slightly to 14.1° ± 6.2 at midterm follow-up. Although no differences were seen regarding baseline data, clinical outcomes, and complications, fracture level significantly altered the COMI score at 24 months with no effect on pain score or quality-of-life.

**Conclusion:**

MIHS using polyaxial screws is a safe treatment for single-level osteoporotic spinal fractures. Fracture level did not alter radiological reduction loss; however, it significantly altered patients’ function at 24 months.

**Supplementary Information:**

The online version contains supplementary material available at 10.1007/s00068-024-02645-1.

## Introduction

Incidences of osteoporotic vertebral fractures (OVFs) rose markedly over the last two decades and OVFs are nowadays a leading cause of morbidity in the elderly [1, 2]. Suboptimal post-traumatic restoration of spinal alignment is a risk factor for additional spinal fractures [3]. Therefore, the target of surgical therapy is two-fold. First, it aims to achieve significant pain reduction. Second, it aims to improve spinal alignment.

Although therapeutic aims are clearly defined, treatment guidelines differ between institutions. The osteoporotic fractures (OF)-classification allows for the extraction of classification-adjusted treatment recommendations and is frequently utilized in Europe. Condensed, conservative therapy is indicated in patients with acute stable OVFs (OF types 0, 1, 2, and type OF 3 without relevant posterior cortex involvement) [4, 5]. In the case of persistent pain, impaired mobility, or increased kyphotic misalignment, minimal invasive cement-augmented therapy strategies are recommended. [6–8].

The current study focussed on unstable OVFs with posterior edge involvement (types OF 3 with pedicle involvement and OF 4). These fracture morphologies are prone to result in kyphotic deformities and subsequent spinal stenosis Intralesional stand-alone procedures such as kyphoplasty or vertebroplasty are believed to be insufficient to adequately restore spinal alignment and stability. Dorsal stabilization is frequently applied for these unstable OVFs. Short-segment posterior stabilization, without anterior support, is associated with hardware failure, loss of reduction, and the development of a postoperative kyphotic deformity. Long-segment posterior stabilization, on the other hand, seems to be too invasive for the geriatric population. More recently, successful minimal invasive hybrid stabilization (MIHS) techniques, including additional cement augmentation of the injured vertebra have been successfully introduced in patients with OVFs and without neurological deficits. [4, 5, 9]. Different modifications of MIHS for OVFs have been described in the literature. Both cement-augmented screws and cementless screws have been utilized, and the short-term clinical outcome is promising [10–14]. However, cemented screw augmentation can lead to complications, such as cement leakage [15], pulmonary embolism [16], and increased risk of infection [17].

To date, midterm (defined as 24-month) radiologic outcomes of MIHS and the impact on quality of life are currently unclear. In our hospital, double treated pedicle screws and titan rods are used to prevent multilevel cementation in the elderly. The current study was performed to determine midterm clinical and radiological outcomes of MIHS with double-threated uncemented pedicle screws for unstable osteoporotic vertebral fractures (OVFs).

## Methods

### Study design and ethical approval

A prospective cohort study was performed at the Department of Spine Surgery at the Rhein-Maas Clinic, an accredited spine center certified by the German Spine Community (DWG). Patients were enrolled in the study between November 1st, 2015, and January 1st, 2018. The protocol was approved by the regional Ethics Committee (file number: 2016448). Informed consent was obtained from all participants.

### Cohort

All patients admitted to our institution for potential spinal injuries were examined clinically and received conventional radiographs. A routine magnetic resonance imaging (MRI) was performed in those patients without contraindications for MRI. Additionally, computer tomography (CT) was carried out in patients with moderate- and high-energy traumas and those individuals with contraindications for MRI. Spinal fracture stability was assessed using the Osteoporotic Fracture (OF) classification system [9]. The following inclusion criteria have been applied:

(1) age > 60 years, (2) a single-level unstable fracture, (3) a fracture at the level of the thoracic or lumbar spine, (4) fracture caused by low-energy trauma with osteoporosis, (5) a type OF 3 with pedicle involvement and 4 according to the OF classification [9].

Of note, according to our protocols, spine tissue sampling was performed and histological analysis was performed in all patients to rule out metastatic origin and to confirm local osteoporotic disease. Meanwhile, cases with metastatic as well as old fractures were excluded from the analysis.

### Surgical technique

All patients were treated with MIHS. This procedure entails a kyphoplasty of the fractured vertebral body and fixation of the adjacent level above and below the fractured vertebral body with uncemented pedicel screws. In short, all interventions were performed under general anesthesia and in a prone position. A balloon-kyphoplasty is performed first. Subsequent minimal invasive pedicle-screw stabilization was done using dual-threated polyaxial screws without cement augmentation (Longitude II, Ballon Kyphon, Fa. Medtronic, Minneapolis, MN, USA). A detailed description of the protocol is attached as Appendix 1.

### Postoperative management and follow-up

After surgery and mobilization, post-operative radiographs in a standing position were made in two planes. An additional CT scan was only made to control for correct screw placement or anatomic reduction in symptomatic patients.

Clinical and conventional radiological assessment was performed prior the intervention, after 3 months, and after 24 months of follow-up. Furthermore, anti-osteoporotic therapy was initiated directly after hospitalization.

### Baseline parameters

The following parameters were collected:

Patient characteristics: gender, age, pre-operative ASA (American Society of Anesthesiologists)-score [18], and body mass index (BMI).

Injury characteristics: fracture classification and specific radiological fracture features, such as kyphosis angles, as described by Cobb, were independently determined by three investigators (MM, AA, and MT) using sagittal conventional X-ray imaging [19–21].

The Alpha Cobb-angle was determined by drawing lines parallel to the upper endplate of and the lower endplate of the fractured vertebral body. The angle between both lines reflected the Alpha Cobb-angle.

The Beta Cobb-angle was calculated by drawing lines parallel to the upper endplate of the vertebral body adjacent to the fractured and the lower endplate of the fractured vertebral body. The angle between these two lines was the Beta Cobb-angle.

### Clinical outcome parameters

Both in-hospital and follow-up outcome data were collected and compared over time.

The following parameters were measured: operative time, estimated intra-operative blood loss, the need for blood transfusion therapy, the need for operative re-intervention/revision surgery [22], length of hospital stay (LOS), and complication rates (defined as Clavien-Dindo Grade 3 or higher) [23]. Examined complications included pneumonia, material outbreak, adjacent spine fractures, clinically relevant cement leakage, and screw loosening. Of note, the diagnostic criteria for screw loosening, in line with the literature, are partly subjective; however specific signs include:

- A radiolucent area (> 1 mm) around the screw [14/4–9].
- Presence of the 'double halo' sign [14/4,10].

Pain and functional outcomes were also determined at 24 months of follow-up. Additional stratification of these midterm outcomes was performed to determine the impact of fracture localization. Three subgroups were investigated: thoracic fractures (fracture above the vertebral body Th11), thoracolumbar fractures (the involvement of the vertebral body Th11-L2), and fractures of the lumbar spine (L3-5). The following parameters were determined and compared:

Visual Analog Scale (VAS): pain assessment was performed using an 11-point numeric rating scale, where zero indicates no pain, and 10 indicates the most intense pain possible [24].

The Core Outcome Measures Index (COMI)-back: a brief instrument for assessing the main outcome in patients with back problems (pain, function, symptom-related well-being, quality of life, and overall disability) [25]. The COMI-back was developed during the 'Spine Tango'-project and is a validated outcome assessment tool for patients with back problems. A higher COMI-back reflects an inferior outcome [26].

European Quality of Life-5 Dimensions (EQ-5D)-score: represents a validated questionnaire-based quality-of-life assessment tool. The EQ-5D consists of different domains determining a respondent’s current health status. Mobility, self-care, activities, pain/discomfort, and anxiety/depression were scored [27, 28].

### Histopathological data

**Fig. 1:**
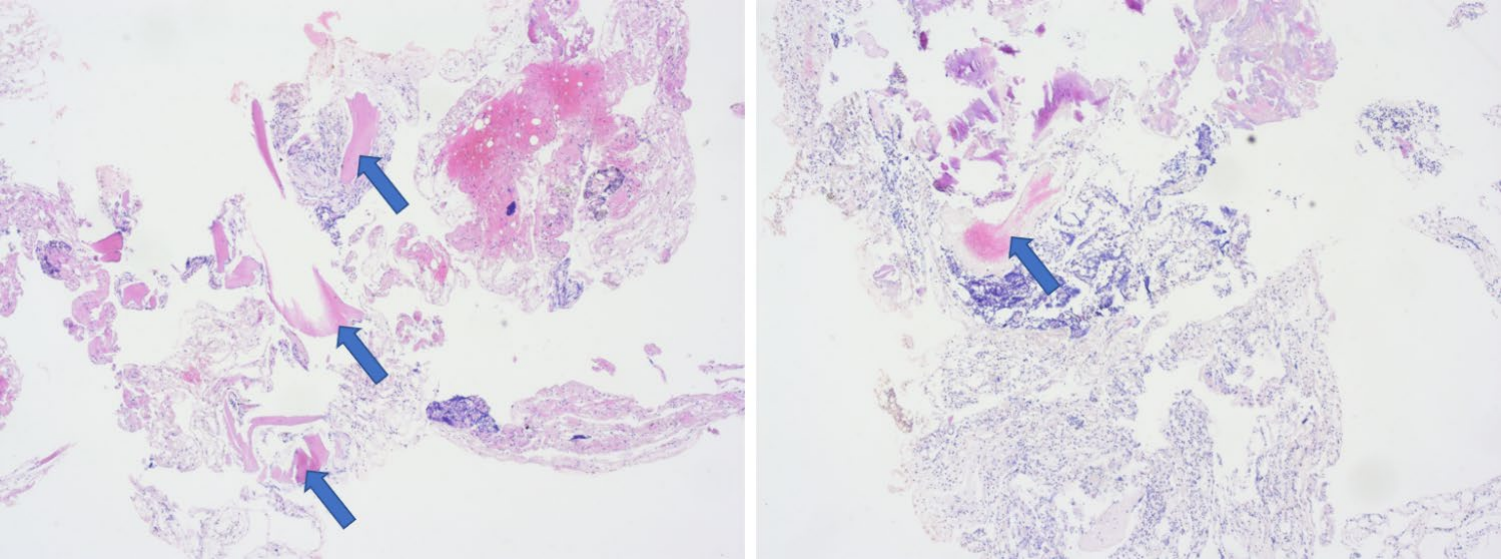
Two histopathological tissue samples show changes consistent with osteoporotic changes in the vertebral spine. The arrows point to cancellous trabeculae with partially fibrotic marrow in between

Osteoporotic changes in included patients were confirmed by the histopathological examination which showed cancellous trabeculae in between there is bone marrow with blood formation cells (blue dots) and fat cells (white holes). Figure 1 shows two tissue samples consistent with these changes. Although very compressed, fragmented, and pushed together with artifacts, bleeding and signs of tissue remodeling were evident by the partially fibrotic marrow in between the cancellous trabeculae.

### Radiologic outcome parameters

The following radiological parameters were studied over time (pre-operatively, post-operatively, and after 24 months):

- Changes in Kyphosis angles [19–21].
- Changes in the Alpha Cobb-angle.
- Changes the Beta Cobb-angle.

### Statistical analysis

Statistical analysis was performed using SPSS 22.0 for Windows (Chicago, IL, USA). A descriptive summary of baseline demographic data was provided in the form of mean (standard deviation) for continuous data and numbers (percentages) for binary/categorical data. For skewed data, the median and interquartile range (IQR) were provided. The differences between groups were calculated using chi-square or Fisher’s exact test for the ordinal data. Either paired or unpaired T-tests or the Mann-Whitney U test were used to analyze continuous data, depending on the assumption of normal distribution. When comparing clinical and outcome data in different spinal fracture levels (thoracolumbar, thoracic, and lumber), the one-way analysis of variance (ANOVA) test was used for continuous outcomes. P-values < 0.05 were considered statistically significant.

## Results

### Patient and fracture characteristics

Patients’ characteristics are shown in Table 1. A total of 73 patients were included. As anticipated, female predominance was seen as 52 (71.23%) female and 20 (28.77%) male individuals were included. Patients had a mean age of 79.9 ± 8.8 years. Patients had an overall preoperative mean ASA-score of 3 ± 0.57 and a mean BMI of 28.0 ± 5.20 kg/m^2^. Most patients were diagnosed with thoracolumbar fractures (*N* = 52, 71.23%), while isolated lumber and thoracic spinal fractures were seen in 13 (17.81%) and 8 (10.96%) patients, respectively. Patients had a mean preoperative alpha-angle of 13.7°± 6.5 and a beta-angle of 16.3°± 7.5. Stratification of baseline data based on fracture level did not reveal any significant changes between groups in terms of age, gender, ASA score, BMI, OF class, and radiological data.

**Table 1:** Baseline characteristics of included patients with osteoporotic vertebral body fracture

Variable	Total	Spinal fracture level			*P*-value
		Thoracic (*N* = 8)	Thoracolumbar (*N* = 52)	Lumbar (*N* = 13)	
Gender (F:M)	52:21	5:3	40:12	9:4	0.215
Age (in years); mean (SD)	79.9 (8.8)	79.76 (11.42)	81.09 (7.57)	84.96 (6.13)	0.105
BMI; mean (SD)	28.00 (5.20)	25.2 (5.75)	26.61 (4.91)	27.84 (5.99)	0.603
ASA-score; mean (SD)	3 (0.57)	2.63 (0.56)	2.77 (0.44)	2.69 (0.54)	0.586
OF classification (OF3: OF4)	47: 26	6:2	33:19	8:5	0.573
Radiological characteristics					
Alpha-angle (preoperative)	13.7 (6.53)	15.63 (7.25)	14.30 (6.44)	7.01 (1.56)	0.184
Beta-angle (preoperative)	16.3 (7.50)	18.41 (9.49)	16.53 (6.74)	11.78 (10.46)	0.558

*F:M* Number of female to male patients; *BMI* body mass index; *IQR* interquartile range; *SD* standard deviation; *ASA* American Society of Anesthesiologists; *N* number of patients per group

### Operative data

Operative time varied between 60 and 120 min in 54 (73.97%) patients and was less than one hour in 14 (19.17%) cases. Prolonged interventions (> 120 min) were reported in five (6.84%) patients, of whom one (1.37%) intervention took more than three hours. A total of 292 pedicle screws were placed in 52 patients. Intra-operative blood loss was < 100 ml in 35 patients (47.94%), 100–500 ml in 36 (49.31%) patients, and > 500 ml in two (2.74%) patients.

Table 2 summarizes operative time and intraoperative blood loss stratified by the level of spinal fracture. No significant differences were noted in operative time (*P* = 0.095) and blood loss (*P* = 0.454) based on the level of spinal fracture.

**Table 2:** Operative time and intraoperative blood loss stratified by spinal fracture level

Variable	Total	Spinal fracture level			*P*-value
		Thoracic (*N* = 8)	Thoracolumbar (*N* = 52)	Lumbar (*N* = 13)	
Operative time (mins); number					
< 60 min	14	0	14	0	0.095
60–120 min	54	7	36	11	
120–180 min	4	1	2	1	
> 180 min	1	0	0	1	
Intraoperative blood loss (ml); number					
< 100 ml	35	5	24	6	0.454
100–500 ml	36	2	27	7	
> 500 ml	2	1	1	0	

*N* Number of patients per group

### Clinical outcomes and complications

In-hospital mortality did not occur and 39 (53.42%) patients had an uncomplicated clinical course. Eleven (15.07%) patients suffered from spine-associated complications and 27 (36.98%) individuals had a total of 37 relevant general complications. Urinary tract infection (*n* = 12), pneumonia (*n* = 5), and electrolyte disturbance (*n* = 9) were the most frequent complications. No cases of neurological impairment or cement leakage were observed. A mean LOS of 13.38 days (SD: 7.20) was reported. Clinically relevant screw loosening was observed in 5 (1.7%) of 292 implanted screws, all of whom required revision surgery. Furthermore, in four (5.48%) patients, secondary adjacent fractures were diagnosed during the follow-up period. Clinical outcomes are shown in Table 3, and complications are displayed in Table 4. Importantly, no significant difference in either clinical outcomes or complications were observed between groups according to the level of spinal fracture.

**Table 3:** Clinical outcomes stratified by spinal fracture level

Variable	Total	Spinal fracturelLevel			*P*-value
		Thoracic (*N* = 8)	Thoracolumbar (*N* = 52)	Lumbar (*N* = 13)	
LOS (days): mean (SD)	13.38 (7.20)	14.9 (8.89)	13.58 (7.42)	11.46 (4.52)	0.093
Prolonged LOS; (%)	0%	0%	0%	0%	0.995
Neurological impairment; (%)	0%	0%	0%	0%	0.995
Significant cement leakage; (%)	0%	0%	0%	0%	0.995
Number of placed screws/loosening (%)/total number of screws	5 (1.7%)/292	0 (0%)/292	4 (1.37%)/292	1 (0.34%)/292	0.481
Number of adjacent fractures; (%)	4 (5.5%)	0 (0%)	4 (5.5%)	0 (0%)	0.554

Prolonged LOS was defined as the number of patients with a length of hospital stay above the 75th percentile

*LOS* Length of hospital stay; *SD* standard deviation

**Table 4:** General complications (Clavien-Dindo grade ≥ 3) stratified by spinal fracture level

Complication	Total	Spinal fracture level			*P*-value
		Thoracic (*N* = 8)	Thoracolumbar (*N* = 52)	Lumbar (*N* = 13)	
Urinary tract infection	12	1	9	2	0.812
Pneumonia	5	1	3	1	0.8
Electrolyte disturbance	9	0	8	1	0.241
Acute kidney failure	4	0	3	1	0.65
Postoperative anemia	3	1	2	0	0.46
Infection of unknown origin	2	1	1	0	0.256
Ileus	2	0	2	0	0.671
Cardiac arrhythmia	1	0	1	0	0.671
Total number of complications	38	4	29	5	0.091

*N* Number of patients

### Radiological outcomes

The alpha-angle improved significantly after intervention with a mean of 5. 4° (4.1). No significant reduction loss (as determined by the alpha-angle changes) was encountered up to 24 months postoperatively (*P* = 0.18). Beta angles also improved significantly after surgery from 16.3 (7.5) to 10.8 (5.6). Midterm radiological follow-up demonstrated a significant increase in beta-angles with a value of 14.1 (6.2). Of note, no significant differences in the alpha and beta angles were observed between different spinal fracture levels at all follow-up time points (Table 5).

**Table 5:** Radiological alterations over time, stratified by spinal fracture level

Variable	Timing	Total	Spinal fracture level			P-value
			Thoracic (N = 8)	Thoracolumbar (N = 52)	Lumbar (N = 13)	
Alpha angle	Preoperative	13.7 (6.53)*	15.63 (7.25)	14.30 (6.44)	7.01 (1.56)	0.184
	Postoperative	8.24 (5.1)*	7.08 (4.44)	9.23 (5.18)	3.51 (1.57)	0.277
	24 months	9.44 (4.5)	7.42 (4.16)	10.76 (4.07)	3.79 (1.86)	0.517
Beta angle	Preoperative	16.3 (7.51)**	18.41 (9.49)	16.53 (6.74)	11.78 (10.46)	0.558
	Postoperative	10.8 (5.6)**/***	11.04 (5.33)	9.81 (3.92)	7.90 (6.38)	0.539
	24 months	14.1 (6.32)***	21.04 (7.05)	14.63 (5.28)	9.83 (7.71)	0.66

All data are reported as mean ± standard deviation

*mo* Month

*P < 0.001, **P < 0.0001, ***P = 0.02. P-value indicates the difference between different spinal fracture levels at each follow-up point using the one-way ANOVA test

### Midterm follow-up: functionality, pain, and quality of life after MIHS

After 24 months of follow-up, a COMI-back of 4.71 (2.65) was observed. VAS-back was higher than VAS-legs with values of 3.88 (2.39) vs. 2.5 (2.92), respectively. An EQ-5D-score of 0.57 (0.34) was found in the cohort. As displayed in Table 6, COMI-back was significantly higher in patients with fractures of the lumbar spine than in patients with more cranial fracture localizations.

**Table 6:** Fracture localization and midterm functional outcome/pain score

	Thoracic fracture (*N* = 8)	Thoracolumbar fracture (*N* = 52)	Lumbar fracture (*N* = 13)
COMI-back	3.00 ± 2.22*	4.69 ± 2.71**	7.13 ± 0.49*/**
VAS-legs	1.25 ± 1.50	2.58 ± 3.15	3.67 ± 2.89
VAS-back	3.75 ± 1.89	3.63 ± 2.54	5.67 ± 1.52
EQ-5D	0.87 ± 0.22	0.78 ± 0.33	0.73 ± 0.37

All data are reported as mean ± standard deviation

*VAS* Visual analogue scale; *EQ-5D *European quality of life-5 dimension; *COMI* core outcome measurement index

*P < 0.05 (thoracic vs. lumbar), **P < 0.05 (thoracolumbar vs. lumbar)

### Clinical example

To illustrate the application and outcomes of the minimally invasive hybrid stabilization (MIHS) with double-threaded uncemented polyaxial screws, we present the following clinical case. A 76-year-old female patient presented with severe back pain following a minor fall. Radiological evaluation, including MRI and CT scans, revealed an unstable osteoporotic vertebral fracture classified as OF 4 involving the thoracolumbar region. The patient had a history of osteoporosis and was previously managed conservatively for similar issues. The patient underwent MIHS using double-threaded uncemented polyaxial screws. Under general anesthesia and in a prone position, a balloon-kyphoplasty was performed to restore vertebral height. This was followed by the fixation of the adjacent level above and below the fractured vertebra with double-threaded polyaxial screws (Longitude II Ballon Kyphon, Medtronic, Minneapolis, MN, USA). The screws were polyaxial and double-threaded (45 mm with cobalt-chromium alloy; Φ 6.5 mm) that provide better anchorage in cortical bone to reduce risk of implant loosening (Supplementary Fig. 1).

The procedure was completed in 90 min with minimal blood loss (< 100 ml). Intraoperative fluoroscopy confirmed appropriate placement and alignment of the screws and rods. Postoperative standing radiographs and CT scans showed satisfactory alignment and no evidence of screw loosening or cement leakage. The patient was mobilized on the second postoperative day and discharged home on postoperative day 10 with significant pain relief. At the 24-month follow-up, the patient reported a significant improvement in functionality and quality of life, with a VAS-back score of 2 and a COMI-back score of 3. Postoperative radiographs maintained the correction of kyphotic deformity with stable alpha and beta angles.

Noteworthy, for low-energy trauma cases, during initial evaluation, x-ray (showing fracture), CT, and MRIs (if T2 oedema in the vertebral body was noted, this indicated fresh/acute fracture) were done. After fixation and during the postoperative period, follow-up CT scans were done (X-ray was not routinely performed) to measure the alpha and beta angles.

## Discussion

The current study is the first to demonstrate that MIHS with double-threated, uncemented screws for unstable osteoporotic vertebral fractures (OF 3 and 4) is associated with (1) adequate post-operative improvement of kyphosis as well as proper 2-year radiological follow-up results, (2) satisfactory midterm functional outcome, pain scores, and quality of life, and (3) low rates of spine-related complications, despite many systemic complications in this investigated geriatric population. Therefore, MIHS with double-treated pedicle screws should be considered a safe and feasible treatment concept for unstable OF-3 and 4 vertebral fractures. This paves the way for more widespread implementation of this cementless pedicle screw technique in the treatment of unstable OVFs.

Different surgical strategies for unstable OVFs have been described in the literature, and treatment guidelines differ between institutions [4, 5], with MIHS being the most commonly used surgical strategy (Table 7) based on the results of the EOFTT study [29]. In our institution, MIHS is considered as the treatment of choice for all patients suffering from unstable OVFs (defined as OF 3 with pedicle involvement and OF 4). Given the limitations of the OF-score for higher-grade injuries, it is not routinely performed nor integrated into our treatment guidelines [9]. Importantly, OF 5 fractures are generally treated with long-segment stabilization constructs.

**Table 7:** A summary of similar original research papers discussing the use of the hybrid stabilization method in osteoporotic vertebral fracture

Author (YOP)	Country	Design	Inclusion criteria	Exclusion criteria	Sample	Surgical protocol	Follow-up (mo)
Our study	Germany	Prospective cohort	Thoracolumbar vertebral fracture (single-level) with OF 3 & 4	Multilevel fractures	73	Balloon kyphoplasty with subsequent minimally invasive pedicle-screw stabilization using dual-threated polyaxial screws without cement augmentation	24
Rerikh (2023) [11]	Russia	Retrospective cohort	Uncomplicated OVB fractures of thoracolumbar spine (T10-L2) either complete or incomplete burst fractures (A3, A4)	Complicated spinal injuries and 2ry osteoporosis	58	Posterior stabilization combined with cement vertebroplasty or osteoplasty	12
					76	Posterior stabilization combined with anterior fusion	
Alhashash (2022) [27]	Germany and Egypt	Prospective cohort	Patients (> 65 years) with OVB fracture of thoracolumbar spine (T5-L5) with OF 3 or 4	Pathological, metastasis-related fractures, OF 5, and critical general condition	45	Anterior approach: Biportal VATS was performed followed by an expandable titanium implant placement to replace the fractured OVB. This was done through a left-sided mini-laparotomy in the supine position for L4-L5, while L2-L3 were operated upon in the lateral decubitus position.	24
						Posterior approach: the prone position, percutaneous bisegmental cannulated screw fixation was performed under the guidance of two perpendicular C-arms. Two short pedicle screws were inserted in the pedicles of the fractured vertebra. The screws in the proximal and distal vertebrae were augmented using high viscosity bone cement. The amount of injected cement depended on the fracture level. In the thoracic area, a maximum amount of 6 ml (3 ml in each screw) was used, and in the lumbar area, a maximum amount of 8 ml was used (4 ml in each screw).	
Spiegl (2019) [13]	Germany	Retrospective cohort	Patients (> 60 years) with acute, unstable OVB fracture of Th10-L4	Subsequent fractures, neurologic impairment, or pathological fractures	113	Hybrid stabilization was done minimally invasive by posterior cement-augmented short-segmental (one level above and one level below the fractured OVB) stabilization without fusion and bilateral transpedicular kyphoplasty of the fractured vertebral body. Pedicles screws were inserted parallel to the superior end plates in Seldinger technique. All pedicle screws were cement augmented.	48
Spiegl (2020) [12]	Germany	Retrospective cohort	Patients (> 60 years) with acute, unstable OVB fracture of Th11-L4	Non-orthograde beam path at the fractured level, neurological impairment, pathological fractures, and high energy trauma	29	Minimally invasive hybrid stabilization by posterior cement-augmented bisegmental instrumentation using polyaxial screws and without posterior fusion and bilateral transpedicular kyphoplasty of the fractured vertebral body. All pedicle screws were cement augmented	36
Spiegl (2018) [14]	Germany	Retrospective cohort	Patients (60–70 years) with unstable OVB fractures (Th11-L3)	Prior or further fractures of the vertebral spine, neurological impairment, and pathological/type C fractures	10	CPAS consisted of posterior stabilization by an open approach using mainly monoaxial implants with cement augmentation of the pedicle screws. Anterior fusion was done by a minimally invasive thoracoscopic approach or by mini-lumbotomy using extandable titanium cages.	27
					19	Hybrid stabilization was performed by minimally invasive techniques using posterior cement augmented, bisegmental instrumentation, and bilateral transpedicular kyphoplasty of the fractured vertebral body.	
Pingel (2014) [10]	Germany	Case report	Burst, OVB fracture at L1		1	Hybrid stabilization: short-segment percutaneous stabilization with cement-augmented screws and kyphoplasty	-
Schnake (2022) [28]	Germany	Multicenter (16 centers) prospective study	Traumatic or insufficiency OVB fractures of thoracolumber spine	History of spinal tumors or infection	368	Kyphoplasty	7
					11	Vertebroplasty	
					25	Posterior stabilization (pedicle screws)	
					11	Posterior stabilization (pedicle screws) with kyphoplasty/vertebroplasty	
					44	Posterior stabilization (pedicle screws) with screw augmentation	
					83	Posterior stabilization (pedicle screws) with screw augmentation and kyphoplasty	
					35	Combined posterior and anterior stabilization	
Spiegl (2023) [29]	Germany	Multicenter (17 centers) prospective study	Spontaneous or low-energy OVB fractures in patients > 18 years of age	–	21	Conservative	6
					16	Cement augmentation only	
					16	Posterior short-segmental	
					31	Hybrid stabilization	
					10	Posterior long-segmental	
					7	Dorso-ventral	

*CPAS* Combined posterior reduction and bi-segmental stabilization followed by additional anterior spondylodesis; *OVB* osteoporotic vertebral body; *mo* month

It is believed that hybrid stabilization procedure optimizes construct stability, without requiring invasive anterior spine surgery. This construct functionally acts as a 360°-stabilization, which is indicated to avoid shear forces in patients with posterior cortex lesions and is associated with promising outcomes in most patients [30–34]. This study further shows that minimal invasive posterior spine surgery for OVFs is associated with short operative time, minimal blood loss, minimal postoperative pain, and short post-operative recovery times. Post-operative restoration of vertebral height in the current study was alike other studies on MIHS for OVFs [23, 32, 35, 36].In addition to others, we managed to further demonstrate adequate 2-year retainment of vertebral height and kyphosis correction.

The safety of MIHS for patients with unstable OVFs in our hands is underlined by the absence of neurological complications and postoperative mortality. Intervention-related complications, however, did occur. In 8 patients irrelevant cement leakage was diagnosed, although no leakage into the medullary canal was seen and no neurological deficits or indications for re-interventions/conversion existed. Screw loosening was diagnosed in 1.7% of screws. To avoid leakage as well as screw malpositioning we recommend probing all pedicles carefully in all quadrants. In addition, the instillation of cement should be performed under fluoroscopic control [32].

Osteoporosis largely affects pedicle screw fixation strength and bone marrow density correlates with pull-out strength [37–39]. To overcome these issues, augmentation of pedicle screws with cement has been introduced already in the 1980s [40]. Fenestrated pedicle screw cement augmentation was approved by the US Food and Drug Administration (FDA) in 2016 and is considered a safe and effective method to increase screw fixation strength in osteoporotic bone. Interestingly, despite the availability of a bulk of evidence on the biomechanical benefits of this technique, few proper studies report its safety and efficiency in a clinical setting [14, 41]. In addition, fenestrated screws are associated with increased epidural cement leakage, without increasing pull-out strength as compared with solid screws in a cadaveric model from Becker et al. [42]. Chen et al. further showed that fenestrated screws have inferior pull-out strengths than regular screws with pre-filling of the vertebra with PMMA [43]. This may be due to suboptimal cement distribution upon administration via fenestrated screws as they also observed that most of the cement left the fenestrated screw from the proximal fenestrations [41, 43]. Cement-augmented screw placement is associated with cement leakage as well. Rates of cement leakage vary between 20 and 93% [37, 38]. A systematic review reported risks of pulmonary embolisms up to 23% [44]. No consensus exists on the treatment of cement extravasation [45].

To prevent cement-related complications in MIHS we decided not to use cement-augmented screws and to focus on optimal design of other components of the fixation construct. To do so, we decided to focus on screw design and rod material. Regarding screw design, we utilized dual lead osteoporotic-specific pedicle screws as these screws demonstrated optimal pull-out properties in multiple biomechanical studies [46–48].

Different types of rod materials are available for spine surgery; however, cobalt-chrome rods are superior to titanium rods in corrective spinal surgery regarding postoperative stability. Therefore, cobalt-chrome rods are favoured in our institution for trauma cases as well [49]. PEEK devices have been used in the past, although due to high failure rates, we decided to phase out these rods before the start of this study [50].

### Strengths and limitations

This study’s strengths lie in its large sample size of geriatric patients, which enhances the generalizability of our results, and the long follow-up period of 24 months, ensuring comprehensive understanding of long-term outcomes. Additionally, the availability of histopathological data enriches our analysis by correlating clinical outcomes with underlying biological mechanisms. These factors contribute to the robustness and clinical relevance of our findings, supporting the potential for MIHS with double-treated, uncemented screws to become a widely adopted treatment strategy for unstable osteoporotic vertebral fractures in elderly patients. However, our study is not without limitations. We were not able to add a control group to our dataset. Consequently, no comparative analysis has been performed. Therefore, upcoming multicentre studies should compare the outcome between different treatment protocols (incl. cemented screws and long-segment stabilizations) and further investigate the impact of spinal segmentation on the outcome. Especially for geriatric patients, in which follow-up studies are hard to perform. Furthermore, we did not compare the quality-of-life and functional outcomes of our patients with other geriatric patients without spinal fractures. Therefore, the natural course of spinal degeneration in elderly individuals was not adjusted for.

## Conclusion

This prospective study is the first to demonstrate that MIHS is a safe and feasible treatment modality for unstable OVFs (OF 3 and 4) in the elderly. Complication rates are low, while radiological outcomes and midterm functional outcomes are satisfactory. The outcome of uncemented double-threated screws further equals the outcome of studies on cemented screws. These findings pave the way for more widespread implementation of MIHS with cementless pedicle screws in the treatment of unstable OVFs in elderly patients.

## Electronic supplementary material

Below is the link to the electronic supplementary material.

Supplementary Material 1 DOCX 21 KB

Supplementary Material 2 TIF 119 KB

## Data Availability

Analyzed data in this research can be provided by the corresponding author upon reasonable request.
