# Supplementary material for: Satisfactory 2-year outcome of minimal invasive hybrid stabilization with double treated screws for unstable osteoporotic spinal fractures
Source: Eur J Trauma Emerg Surg. 2024 Aug 30;50(5):2385–98. doi: 10.1007/s00068-024-02645-1 (PMC11599358; doi:10.1007/s00068-024-02645-1)
Supplement: Supplementary file 1 — Supplementary Material 1 DOCX 21 KB [file 68_2024_2645_MOESM1_ESM.docx]

**Table S1.** A summary of available original research studies investigating the efficacy of hybrid stabilization in patients with osteoporotic vertebral body fractures

| Author (YOP) | Country | Design | Inclusion Criteria | Exclusion Criteria | Sample | Surgical Protocol | Follow-up (mo) |
| --- | --- | --- | --- | --- | --- | --- | --- |
| Our Study | Germany | Prospective cohort | Thoracolumbar vertebral fracture (single-level) with OF 3 & 4 | Multilevel fractures | 73 | Balloon kyphoplasty with subsequent minimally invasive pedicle-screw stabilization using dual-threated polyaxial screws without cement augmentation | 24 |
| Rerikh (2023) | Russia | Retrospective cohort | Uncomplicated OVB fractures of thoracolumbar spine (T10-L2) either complete or incomplete burst fractures (A3, A4) | Complicated spinal injuries and 2ry osteoporosis | 58 | Posterior stabilization combined with cement vertebroplasty or osteoplasty | 12 |
|  |  |  |  |  | 76 | Posterior stabilization combined with anterior fusion |  |
| Alhashash (2022) | Germany and Egypt | Prospective cohort | Patients (>65 years) with OVB fracture of thoracolumbar spine (T5 - L5) with OF 3 or 4 | Pathological, metastasis-related fractures, OF 5, and critical general condition | 45 | Anterior approach: Biportal VATS was performed followed by an expandable titanium implant placement to replace the fractured OVB. This was done through a left-sided mini-laparotomy in the supine position for L4-L5, while L2-L3 were operated upon in the lateral decubitus position. | 24 |
|  |  |  |  |  |  | Posterior approach: the prone position, percutaneous bisegmental cannulated screw fixation was performed under the guidance of two perpendicular C-arms. Two short pedicle screws were inserted in the pedicles of the fractured vertebra. The screws in the proximal and distal vertebrae were augmented using high viscosity bone cement. The amount of injected cement depended on the fracture level. In the thoracic area, a maximum amount of 6 mL (3 mL in each screw) was used, and in the lumbar area, a maximum amount of 8 mL was used (4 mL in each screw). |  |
| Spiegl (2019) | Germany | Retrospective cohort | Patients (>60 years) with acute, unstable OVB fracture of Th10-L4 | Subsequent fractures, neurologic impairment, or pathological fractures | 113 | Hybrid stabilization was done minimally invasive by posterior cement-augmented short-segmental (one level above and one level below the fractured OVB) stabilization without fusion and bilateral transpedicular kyphoplasty of the fractured vertebral body. Pedicles screws were inserted parallel to the superior end plates in Seldinger technique. All pedicle screws were cement augmented. | 48 |
| Spiegl (2020) | Germany | Retrospective cohort | Patients (>60 years) with acute, unstable OVB fracture of Th11-L4 | Non-orthograde beam path at the fractured level, neurological impairment, pathological fractures, and high energy trauma | 29 | Minimally invasive hybrid stabilization by posterior cement-augmented bisegmental instrumentation using polyaxial screws and without posterior fusion and bilateral transpedicular kyphoplasty of the fractured vertebral body. All pedicle screws were cement augmented | 36 |
| Spiegl (2018) | Germany | Retrospective cohort | Patients (60-70 years) with unstable OVB fractures (Th11-L3) | Prior or further fractures of the vertebral spine, neurological impairment, and pathological / Type C fractures | 10 | CPAS consisted of posterior stabilization by an open approach using mainly monoaxial implants with cement augmentation of the pedicle screws. Anterior fusion was done by a minimally invasive thoracoscopic approach or by mini-lumbotomy using extandable titanium cages. | 27 |
|  |  |  |  |  | 19 | Hybrid stabilization was performed by minimally invasive techniques using posterior cement augmented, bisegmental instrumentation, and bilateral transpedicular kyphoplasty of the fractured vertebral body. |  |
| Pingel (2014) | Germany | Case report | Burst, OVB fracture at L1 | | 1 | Hybrid stabilization: short-segment percutaneous stabilization with cement-augmented screws and kyphoplasty | - |
| Schnake (2021) | Germany | Multicenter (16 centers) prospective study | Traumatic or insufficiency OVB fractures of thoracolumber spine | History of spinal tumors or infection | 368 | Kyphoplasty | 7 |
|  |  |  |  |  | 11 | Vertebroplasty |  |
|  |  |  |  |  | 25 | Posterior stabilization (pedicle screws) |  |
|  |  |  |  |  | 11 | Posterior stabilization (pedicle screws) with kyphoplasty/vertebroplasty |  |
|  |  |  |  |  | 44 | Posterior stabilization (pedicle screws) with screw augmentation |  |
|  |  |  |  |  | 83 | Posterior stabilization (pedicle screws) with screw augmentation and kyphoplasty |  |
|  |  |  |  |  | 35 | Combined posterior and anterior stabilization |  |
| Spiegl (2023) | Germany | Multicenter (17 centers) prospective study | Spontaneous or low-energy OVB fractures in patients >18 years of age | - | 21 | Conservative | 6 |
|  |  |  |  |  | 16 | Cement augmentation only |  |
|  |  |  |  |  | 16 | Posterior short-segmental |  |
|  |  |  |  |  | 31 | Hybrid stabilization |  |
|  |  |  |  |  | 10 | Posterior long-segmental |  |
|  |  |  |  |  | 7 | Dorso-ventral |  |

CPAS: combined posterior reduction and bi-segmental stabilization followed by additional anterior spondylodesis; OVB: osteoporotic vertebral body; mo: month.
