# Supplementary figures and images for: Satisfactory 2-year outcome of minimal invasive hybrid stabilization with double treated screws for unstable osteoporotic spinal fractures
Source: Eur J Trauma Emerg Surg. 2024 Aug 30;50(5):2385–98. doi: 10.1007/s00068-024-02645-1 (PMC11599358; doi:10.1007/s00068-024-02645-1)

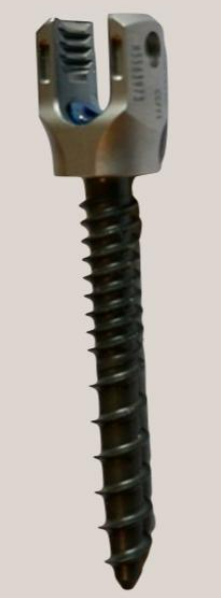

Supplement: Supplementary file 2 — Supplementary Material 2 TIF 119 KB [file 68_2024_2645_MOESM2_ESM.tif]
